# Perception of Climate Change in Shrimp-Farming Communities in Bangladesh: A Critical Assessment

**DOI:** 10.3390/ijerph16040672

**Published:** 2019-02-25

**Authors:** Shaikh Mohammad Kais, Md Saidul Islam

**Affiliations:** 1 Department of Sociology, University of Rajshahi, Rajshahi 6205, Bangladesh; 2 Division of Sociology, Nanyang Technological University Singapore, Singapore 639818, Singapore; msaidul@ntu.edu.sg

**Keywords:** Popular discourse of climate change, radical approach, Bangladesh, industrial aquaculture

## Abstract

Local contexts as well as levels of exposure play a substantial role in defining a community’s perception of climate and environmental vulnerabilities. In order to assess a community’s adaptation strategies, understanding of how different groups in that community comprehend climate change is crucial. Public risk perception is important as it can induce or confine political, economic, and social actions dealing with particular hazards. Climate change adaptation is a well-established policy discourse in Bangladesh that has made its people more or less aware of it. Similarly, shrimp-farming communities in southwestern Bangladesh understand environmental and climate change in their own ways. In order to understand how the shrimp-farming communities in coastal Bangladesh perceive current climate instabilities, we conducted a qualitative study in shrimp-farming villages in coastal Bangladesh where about 80% of commercial shrimp of the country is cultivated. We compared farmers’ perceptions of local climate change with existing scientific knowledge and found remarkable similarities. Our assessment shows that at least two factors are critical for this outcome: coastal people’s exposure to and experience of frequent climate extremes; and a radical approach to defining climate regimes in Bangladesh by various stakeholders and the media, depicting anthropogenic global warming as a certainty for the country. Thus, a convergence of scientific construct and sociocultural construct construes the level of awareness of the general public about climate change.

## 1. Introduction 

Against the backdrop of increasing certainty that human contribution to global warming is decisive, it is believed that the current trajectories of climate anomalies will aggravate existing vulnerabilities and have striking repercussions for natural and social systems around the globe [1]. With the scorching fact that 2018 ranked among the four hottest years on record globally alongside 2015, 2016 and 2017 [2] and with the labelling of global warming as ‘weapon of mass destruction’ of the time [3], climate change is now a major political issue at national and global politics [4,5] that draws attention from science, society, and the media [6,7,8]. As such, the geopolitics of fear leads the ‘doomsday clock’ of the *Bulletin of the Atomic Scientists* to recognize climate change as one of the key threats along with nuclear and biosecurity risks, which can bring severe challenges to humanity [9]. Moreover, climate change has gradually been politicized and mediatized since the late 1980s in both developed and developing nations [6,10,11]. An inconsistent but growing interest of the media in the issue has led to an increased level of understanding among lay people from all sectors including aquaculturists in coastal areas around the global South that again include shrimping communities in Bangladesh. 

Industrial shrimp, locally known as ‘white gold’ [12] because of its high transnational value, is a ‘multimillion dollar industry in Bangladesh’ [13]. Bangladesh is the sixth contributor in global aquaculture [14], supplying about 5% of global shrimp production [15]. This sector directly or indirectly benefits nearly 15 million people [16] while providing subsistence directly to 1.2 million people in Bangladesh [17]. The growth of this sector is retarded by environmental and climate change phenomena, among others. Coastal Bangladesh, where shrimp is cultured, is frequently affected by extreme climate events like cyclones and storm surges that severely damage the entire coastal aquaculture [18]. Bangladesh, as a whole, is one of the most vulnerable countries in the world to climate risks, a fact that made it a hotspot in the world [19]. Climate change vulnerability of the country lies in the fact that two-thirds of the country is less than 5 meters above sea level [20] and is projected to lose 17.5% of its land if sea level rises about 1 meter [21], thereby displacing millions of people. Based on data from the 1980–2000 period, Bangladesh was identified by the United Nations Development Programme (UNDP) as the most vulnerable country in the world to tropical cyclones [22] and the sixth most vulnerable country to floods [23]. Every three years on average, a severe cyclone hits coastal Bangladesh [20,24]. Cyclone Sidr alone resulted in damages and losses of about USD 1.7 billion, or 2.6% of the GDP in 2007 [20]. Super Cyclone Sidr hit the Bangladesh coastal line in November 2007 with a sustained wind speed up to 250 km/h and storm surges up to 9.1 meters, affecting livelihoods of around 8.9 million people with a death toll of 3406 [25,26]. The same cyclone washed away about 54,000 shrimp farms and hatcheries in the coastal districts [25]. 

Cultural and individual preconceptions shape human perceptions of a socioenvironmental issue. Similarly, local contexts and level of exposure play a considerable role in defining a community’s perception of climate and environmental hazards. In order to assess a community’s adaptation strategies, understanding of how different groups in that community comprehend climate change is vital. Public risk perceptions can necessarily induce or confine political, economic and social action to deal with particular risks. If a household or community apprehends shifts in weather parameters, it can take measures to adapt to new conditions, or it can search for supports from state and other stakeholders. The ambiguity of climate change risks is that they are sometimes referred to as ‘dread risks’—in the sense that they are hard to control, have potential for disasters, and permeate the feelings of dread—or as ‘unknown risks’, since slow onset change in climate is hard to readily observe or quantify [27]. Thus, how individuals living in the hazardous zones perceive the gradual changes in climate and weather parameters is very critical. 

Drawing on above narratives, this paper reports on a risk perception study that was carried out to gain an understanding of commercial shrimpers’ perception of climate change impacts on the industry in coastal Bangladesh. We also analyze the factors that contribute to specific understanding of weather and climate in the research areas. Section 2 discusses the two conceptual lenses that frame this study: climate risk perception, and media representation of climate change. Section 3 describes the methods of data collection for this study. In Section 4, we provide an examination of how shrimping communities of Bangladesh understand the recent changes in weather and climate. In Section 5, we try to assess why there are similarities between local people’s perceptions and scientific data on climate change in Bangladesh. We conclude in Section 6 by summarizing our findings and showing their broader implications. 

## 2. Conceptual Framework

The study has been framed by two conceptual threads: perception of climate risks, and media representation of climate change.

### 2.1. Perception of Climate Risks

From a biopsychological point of view, human perception is the final part of a complex psychophysical process. At first, an information from the environment (e.g., light, sound) activates a person’s sense receptors in sensory organs. Then, through transduction process, the sense receptors convert the physical energy from stimulus into electrical signals called neural impulses and send them to the brain. When neural impulses reach the particular area in the brain, they are changed into meaningless bits of information called sensation, which involves the detection of sensory stimuli. These meaningless bits of information are finally changed into meaningful and complete images called perception—the interpretation of sensory stimuli [28,29,30,31,32]. Prior knowledge and experience help the brain interpret a certain stimulus positively or negatively. This fundamental process is at the root of human understanding of environmental big issues like global warming. 

Studies of human behavior indicate that the performance of a behavior is “a joint function of intentions and perceived behavioural control” [33]. Thus, the link between how people perceive risk and then act on it is a construct. Cultural adherence and social learning can frame the perception of climate change risks [27]. Boholm (1998) notes in this context, “Actions and understandings about risks… are informed by socially and culturally structured conceptions and evaluations about the world, what it looks like, what it should be or should not be. Perceptions of events and phenomena are conditioned by values, which vary according to local bodies” [34]. This implies that people from different cultures may perceive climate risks differently. Thus, cultural theory of risks can foresee and explain ‘what kind of people will perceive which potential hazards to be how dangerous’ [35]. 

Similarly, social location of an individual can determine their vulnerability and exposure to risks, which in turn influence their perception. Social location is defined by a person’s job, income, race, ethnicity, gender, religion, political ideology etc. [36] Nursey-Bray and colleagues (2012) point out that factors that influence the interpretation of a risk include the level of knowledge, the probability of harm, the ability to contend with or mitigate the risk and the value of the resource at risk [27]. 

### 2.2. Media Representations of Climate Change

Both mass and interpersonal communications play pivotal roles in building public perception of risk [37]. The concept of ‘framing’, how information is presented to others [38], is of particular significance to social scientists. Consequently, media coverage of risk has become an important topic of risk communication studies developed in the last three decades [6]. Several studies have shown that understanding of the causes and consequences of global warming by the lay public depends to some extent on how local media illustrates the problem to them [39,40]. Two contending types of knowledge of climate change are worth mentioning—the scientific construct and the cultural construct [41]. Media play a critical role by building a bridge between scientists and public especially in the latter case. 

Similarly, portrayal of anthropogenic climate change in mass media entails two interrelated issues: climate change as science communication and climate change as risk communication [6]. Compared to news on crime, politics, sports, entertainment and so on, scientific facts are relatively unappealing for media coverage in general. The ‘news value’ of scientific information, however, rises when it involves threat to human life [42] or when it invokes controversies and conflicts instead of consensus among the scientific community [43]. It can be argued that both media and public may have less interest about the issues and events that deal with the complexity of climate science but the same issues can attain significant importance once they convey personal or societal threats. 

Media representation of environmental risks is also influenced by dominant debates on the risks. Giddens (2008) distinguished three different positions of scientists in climate change debates. First, the climate change *skeptics* claim there is no ample evidence that present-day processes of global warming are produced by human activity. They argue that the current fluctuation in climate is produced by natural causes, as were the similar cases in the past. Second, the *mainstream* scientists, led by the Intergovernmental Panel on Climate Change (IPCC), are at consensus about anthropogenic causes of global warming. Ninety seven percent of climate scientists agree that climate change is occurring and is human-made [44]. Third, the *radicals* think climate change is a more urgent and greater threat than is commonly acknowledged. The radicals, who originated from the mainstream bloc, see climate change as a present reality, and not merely a future possibility, since they argue that many parts of the world has already been affected by global warming. Neverla and colleagues (2012) identify a fourth group of scientists whom they call ‘pragmatists’. The *pragmatists* hold that greenhouse gas emissions cause global warming; however, they are unconvinced of mainstream scientists’ predictions about future climate changes, which they believe are often exaggerated in media [6]. 

Neverla and colleagues (2012) argue that climate change risk coverage in the media is subject to several factors including the nature and extent of risk, politicization of the risk, journalistic culture, socioeconomic and geographic conditions, and relevance of the issue to the people [6]. If global warming is not politicized in a country with due importance and if most citizens are not exposed to the threats, lay public’s perception in that country can be skeptic. United States is an example. A 2011 Pew survey finds that only 38 percent of Americans believe that there is solid evidence of human-induced climate change [45]. Likewise, if climate change issues in a country are framed from a radical perspective and viewed as an important matter, mass media along with politicians and the civil society in that country often regard global warming as a day-to-day reality. This is evident in Bangladesh where radical policymakers and NGOs are main parties in climate discourses [6]. 

## 3. Materials and Methods

In order to examine local people’s understanding of the trends in global climate change in coastal Bangladesh, a field-level investigation in Bangladesh was conducted from March to July, 2014 and a follow-up study was conducted in 2017. We collected data through a triangulation methods comprised of content analysis of secondary sources, ethnography, and qualitative interview. We applied multiple methods in our study to gain a comprehensive insight into complex and diverse issues of climatic shifts and human perceptions. To get a full picture of the historical as well as current scenario of the climate change in Bangladesh, at first an ‘unobtrusive’ or ‘nonreactive’ [46] research based on content analysis of existing secondary documents in Bangladesh was conducted. In the next phase, we conducted short ethnographic studies staying two weeks in each of the sites in shrimping areas in three southwestern districts of Bangladesh namely Bagerhat (Mongla subdistrict), Khulna (Koyra subdistrict), and Satkhira (Shyamnagar subdistrict). We selected these districts because of some compelling reasons. First, these three districts comprise about 80% of total shrimp farms in Bangladesh [47,48]. Second, these areas are most vulnerable to climate change events like tropical cyclones, sea-level rise, salinity intrusion, and storm surges [13,23,49,50,51]. Finally, through their daily experiences, people of these areas are aware of the current shifts in climate in the locality. 

At the final stage, after having a clear understanding of the local dynamics through ethnography, we interviewed a section of people from various stakeholders (forty-five persons in total, see Table 1)—comprised of shrimp farmers, fry collectors, and shrimp and fry traders—in order to complement the ethnographic data. In order to conduct an in-depth qualitative study, we set specific selection criteria for the respondents that prioritized experienced participants who have the greater depth of understanding and knowledge of shrimp aquaculture and local climate change. Shrimp cultivators: (1) Residing in the village or locality since their birth, (2) Spent at least 15 years in shrimp farming, and (3) Experienced at least one major climate extreme in their lifetime. Shrimp fry (postlarvae) collectors: (1) Residing in the village or locality since their birth, (2) Spent at least 15 years in shrimp fry catching, and (3) Experienced at least one major climate extreme in their lifetime. Shrimp/PL trader: (1) Spent at least 15 years in shrimp or fry trading, and (2) Experienced at least one major climate extreme during their business years. 

Taken as a whole our triangulation of methods provided us with an in-depth understanding of the local dynamics of and narratives over climate change and a comprehensive accounts of risk perception by local people.

## 4. Climate Change Perception in Shrimping Communities

Climate change is a well-established policy discourse in Bangladesh. Most people are more or less aware about it. Shrimp farming communities in Mongla, Koyra and Shyamnagar also understand environmental and climate change in their own ways. Shrimp farmers consider environmental and climate perturbations—together with disease outbreaks in shrimp, scarcity of disease-free post-larvae, lack of sufficient credit flow, lack of proper knowledge and training, and illegal status of fry catching—as one of the major challenges to the industry [52]. They perceive that changes in climate had occurred within the last 10 to 15 years. One notable point is that their perceptions were influenced by the season and timing of the interviews and the immediate past season. For example, a major part of data collection for this study was conducted in the summer of 2014, so they replied questions on heat waves and temperature changes judging that year’s experience; similarly, they answered questions on changes in winter weather mainly based on their experience of the immediate past winter season. Nevertheless, despite this type of ‘incumbency effect’ of weather, shrimp-farming people strived to compare weather patterns between the present and the past 10–15 years. Overall, aquaculturists from all the three sites have fairly similar opinions on changes in weather/climate parameters and changes in the climate. While the impact of changes in climate and environmental conditions and shocks may be different in line with the level of well-being of the household, there are no differences between them in perceiving the changes, since all households of the three areas tend to witness the same climate phenomena. Table 2 summarizes shrimping community’s perceptions of climate change and compares with scientific evidence. 

**Figure 1:**
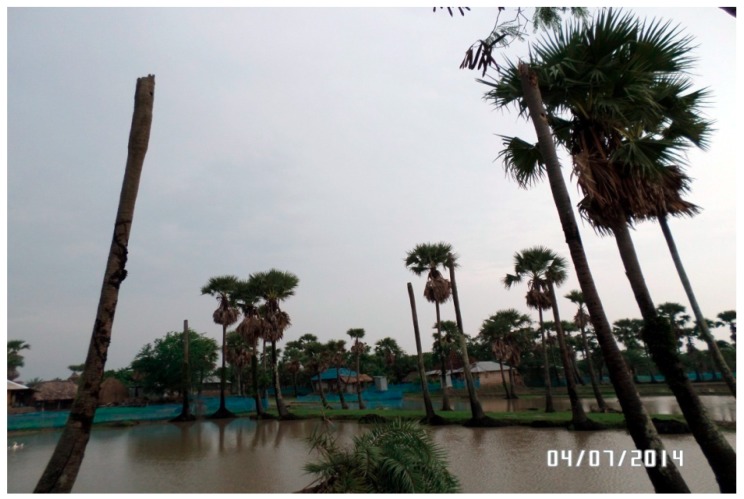
Marker of climate change: Trees die after salinity ingress in Koyra. Source: Field data.

In addition to changes in the above-mentioned primary indicators of weather and climate, people perceive other related changes in the environment. According to their opinions, the number of trees in the vicinity has reduced drastically. Especially after the devastating Sidr and Aila, most of the old trees died because of severe soil salinity. Making landfall during high tide in late May in 2009, cyclone Aila was associated with tidal surges of up to 6.5 meters, affecting 3.9 million people in 11 coastal districts [25,59]. Storm surges engulfed the whole areas overtopping the embankments by side of the rivers and saline water from the sea remained within the polders in whole areas of Koyra and Shyamnagar years after those catastrophes, due to drainage congestion in the polders. During the 1960s, earthen embankments were made along the banks of major rivers under the Coastal Embankment Project in the then East Pakistan in order to protect the reclaimed land from saline tidal surges and to enhance agricultural production in the area [60,61]. These reclaimed and engineered landmasses are called polders. Bangladesh Water Development Board (BWDB) constructed 139 polders in southern Bangladesh [57]. Local people opined that the total number of trees in the area has reduced to one-fourth comparing to that before Aila (see Figure 1). One shrimp farmer from Koyra sighed, ‘Aila destroyed the history of the past 100 years’, referring to the loss of vegetation in the area. Aila wiped out most of the fruit trees in the affected areas including mango, banana, jackfruit, litchi etc. Farmers from Mongla reported that the fruit trees, which grew well in coastal areas previously like coconut and areca, are now struggling to survive. After Aila, local people attempted to plant trees, but those attempts mostly failed because of excessive salinity in water and soil. According to them, most of the tree species died just a couple of days after planting, especially for the first three years after Aila. For the last couple of years some newly planted trees are surviving. Until now, fruit trees’ survival rates are low, only saline tolerant species like caraway grow well. 

Another indicator of environmental change that influences people’s perception is that the production of crops decreased significantly due to increase in water and ground salinity. Some people try hard to cultivate rice in their fields, who would cultivate rice before, but they get very low yields even after applying pesticides and fertilizers.

Raising livestock has become difficult. Grazing land has reduced. Though a portion of existing livestock died or was wounded during Sidr and Aila, the major setback came from the shortage of their food. Grassland reduced, vegetation hampered, and rice cultivation halted—all these lead to a significant decrease in livestock in the areas; local people link these phenomena with climate change.Aquaculture community also observed more insects and pests in crops, vegetables, and fruit trees. The people who grow fruits and vegetables on highlands and home yards for some time in a year reported frequent pest attacks. They associate this with shifting pattern in seasons. 

In addition to the above-mentioned environmental and ecosystems markers of climate change, people in the study areas noticed frequent occurrences of several health problems and diseases in humans associated with extreme and uneven patterns of heat, cold and rainfall including dysentery, headache, asthma, fever, and cough.

## 5. Explaining Similarities Between Perceptions and Scientific Data

People had previously experienced shattering natural disasters like cyclone Sidr and Aila and anticipate more disasters in the future due to a shift in the climate regime. Their understanding of climate variability is essentially drawn from proxy or secondary indicators like crop failure, scarcity of fish in open water, livestock loss, increased sickness as well as the shared experiences of other members of the community. The community does not use any modern technologies to gauge weather changes; rather they attribute sickness, crop failures and other phenomena such as extreme heat or cold events or natural disasters. From Table 1 we find that farmers’ perceptions of climate shifts significantly match with scientific findings. Though the interviewed rural people have little formal education and live on aquaculture and agriculture, they have clear perceptions of the changes in weather parameters that had taken place. Similarly, striking conclusions were drawn by Haque et al. (2012) researching on climate change perceptions of local people in Khulna and Rajshahi [62]. In a study in the shrimp aquaculture community in Mongla, one of the sites of this study, Shameem, Momtaz and Kiem (2015) found a high correlation between scientific evidence and local perceptions of climate change, at least for short-term variability [63]. Other recent studies also conclude that coastal people in India and Bangladesh are aware about the recent changes in local climate [64,65]. 

This research finds two issues that explain the similarities between perceptions of common people and scientific data on climate change in Bangladesh. First, people living in the coastal areas of Bangladesh are among the first victims of climate change in the world. Coastal Bangladesh is an ideal zone for gradual onset changes in climate due to global warming as well as for climate extremes. This is a vulnerable area for sea-level rise (SLR), salinity intrusion, severe cyclones, storm surges, increase of sea surface temperature (SST) and other climate chaos. People living here experience the shifting weather patterns directly through extreme events and indirectly through consequent changes in the quality of water, air, and food as well as changes in human settlements, ecosystems, agriculture, industry, and the economy [62]. They learn about climate change practically by their own experiences. To them, vivid and recent experiences overwhelm abstract statistics about climate change. As we know, human brain is designed to attend to the immediate situation, not out-of-sight data and beyond-the-horizon dangers [66,67]. Therefore, the coastal people can very well map the regime change. Inhabitants of all the three areas live within 70-80 km up-estuary. They regularly monitor the height of tide in the rivers, feel the changes in salinity level in water and soil, and watch catastrophic cyclones and storm surges. The coastal people in Bangladesh, as a vulnerable group, see climate change as something already occurring, rather than something coming in the future. Thus, their perceptions are based on the real changes in the exposed ecosystems—they do not just rely on theoretical narratives. 

Second, as mentioned above, people’s perception of their vulnerability is also shaped by the way it is communicated in the media. Communication, both mass and interpersonal, has an important role in the course of creating public understanding of risks. At the beginning of this century, climate change and global warming became ‘grand narratives’ [6] for science, politics, and the public. As mentioned above, media coverage of climate change is crucial in bridging the gap between scientific conclusions and public understandings. Limited public perception about global climate change in the global North and South, in general, is viewed as a communication problem. The case of Bangladesh is exceptional and crucial. In scientific and policy discourses, the country is frequently termed as one of the worst victims of anthropogenic global warming. Furthermore, climate change is taken not only as an environmental problem but also as an economic concern and development threat in the policy and media discourses in Bangladesh, at least in the last two decades. Thus, in Bangladesh media, global warming is portrayed as a scientific as well as risk phenomenon.

Among the mainstream, skeptic, radical, and pragmatic schools of climate change politics, vast majority of Bangladeshi stakeholders—including scientists, policy makers, governmental institutions, corporate business members, environmental activists, civil society organizations and NGOs alike—take a radical approach in defining and identifying climate regimes in Bangladesh. Bangladeshi media and stakeholders depict anthropogenic climate change as an ‘absolute certainty’ [6] for the country—as if it is an imminent catastrophe. Accordingly, policy discussions in Bangladesh imply that the country has no capacity for the luxury of ‘going slow’ or adopting a ‘wait-to-learn’—it has to take immediate adaptive actions. 

Within this context, different stakeholders venture to transmit climate change messages to the public. Radio, TV, and newspapers frequently publish news reports, features, and articles on human-induced warming of atmosphere. Government and non-government agencies and organizations prioritize climate change agenda in their programmes and projects. Especially NGOs make people aware about likely changes in the climate and devise various coping strategies in their projects, particularly in disaster-prone areas like the south-western coast. For example, we found several NGO projects running in the research areas at the time of field survey for this study. To name a few of the projects: Disaster, Environment and Climate Change (DECC) by BRAC, Increasing Resilience and Reducing Risk of Coastal Communities to Climate Change and Natural Hazards in the Bay of Bengal (Paribartan) By JJS, and Strengthening Livelihood Security of Climate Change Vulnerable People by LEDARS. All the above-discussed factors make local people aware of the changes in weather, climate, and the environment. 

## 6. Conclusions

The evaluation of lay people’s perception is a popularly accepted method to study climate change at the local level [68] and local stakeholders’ awareness of anticipated risks is a crucial contributor to making adaptive changes [69]. The aim of the present research was to generate knowledge on the extent to which shrimp cultivators perceived changes in climate in Bangladesh. Local perception of obvious weather changes was associated with increasing frequency of climate extremes like cyclones and storm surges as well as with temperature increase that they feel and gauge in their own way without the help of any scientific instruments. Although aquaculturists are not much acquainted with the term SLR, they assess the changes by comparing the water level in nearby rivers at present with that of 10–15 years ago. They also perceive the increased level of water and soil salinity by observing the gradual loss of vegetation in the area. They understand changes in climate through perceived negative impacts that the changes might have on local ecosystems, human health, and overall livelihoods. Shrimp farmers are aware about how their lives are affected by the changing and erratic pattern of rainfall, temperature, salinity, and climate extremes. 

By examining coastal people’s understanding and knowledge, this study finds that the shrimpers’ perception of climate change substantially matches to the established scientific data. It reveals several reasons behind this resemblance. Living in an exposed zone for several climate chaos and extremes, people of these areas base their attribution of hydroclimatic phenomena on their repeated personal experiences, on associated impacts on their lives and livelihoods, and on mediatization of global warming as absolute certainty in the country. Thus, a convergence of scientific construct and sociocultural construct construes this level of awareness of the general public.

In a warmer regime, traditional sources of livelihood are seriously threatened and people’s quality of life is in danger of deterioration among vulnerable communities around the globe. In addition to scientific data and grand models, local stakeholders’ perceptions are crucial in determining the changes and variations in local-level weather pattern in different agroecological zones. Appropriate policy and programmatic interventions for exposed communities can be formulated based on the findings of studies on climate change perceptions in those communities. This research is an addition to the systematic collection of such information from a climate hotspot. 

## Figures and Tables

**Table 1:** Selection of respondents.

Participants	Area			Total
	Mongla	Koyra	Shyamnagar	
**Shrimp Farmer**	8	8	8	24
Fry Collector	3	3	3	9
Fry Trader	2	2	2	6
Shrimp Trader	2	2	2	6
Total	15	15	15	45

**Table 2:** Shrimp-farming community’s perception of climate change.

Parameter/Phenomenon	Aquaculturists’ Perception (a)	Scientific Data (Trend) (b)
Summer temperature	Increased heavily.	Average maximum temperature increased by 0.05 °C/year. Average minimum temperature increased by 0.03 °C/year. ^1^
Winter temperature	Overall increased slightly with bitter cold waves for few days.	Mean December temperature increased by 0.23 °C /decade. Mean January temperature increased by 0.05 °C /decade. ^2^
Monsoon rainfall	No change, delayed.	Rainfall increased by 2.24mm/year. ^3^
Winter rainfall	Decreased, virtually no rain.	No significant change. ^4^
Frequency of cyclones or storms	No change.	Increased in the second half of the 20th century. ^5^
Severity of cyclones or storms	Increased slightly.	Increasing trend. ^6^
Flood	Occasionally by storm surges, severe (Aila, Sidr).	25% increase in the number of major floods over the last 100 years. ^7^
Salinity ingression	Heavily increased.	About 6,000 ha of new land affected every year. ^8^
Sea-level increase	About 0.5 m within last 15 years in nearby rivers.	About 4-5 mm/year in the west coast. ^9^
Drought	No or very few droughts in the locality.	Increasing trend in northern Bangladesh. ^10^

Source: (a) Field data; (b) Various secondary sources. 1: [53]; 2: [54]; 3: [54]; 4: [54]. 5: Between 1891 and 2007 a total of 26 severe cyclonic storms crossed the Bangladesh coast, among which 19 occurred in the last 58 years starting in 1960, whereas only 7 occurred in the previous 59 years [55]; 6: [55]; 7: [56]; 8: [57]; 9: [58]; 10: [53].
